# Aggravating effect of abnormal low-density protein cholesterol level on coronary atherosclerotic plaque in type 2 diabetes mellitus patients assessed by coronary computed tomography angiography

**DOI:** 10.1186/s12933-024-02304-0

**Published:** 2024-07-04

**Authors:** Yi-Ning Jiang, Yue Gao, Yu-Shan Zhang, Chen-Yan Min, Li-Ting Shen, Wei-Feng Yan, Zhi-Gang Yang, Rui Shi, Yuan Li

**Affiliations:** Department of Radiology, West China Hospital, Sichuan University, 37# Guo Xue Xiang, Chengdu, 610041 Sichuan China

**Keywords:** Low-density lipoprotein cholesterol, Type 2 diabetes mellitus, Coronary computed tomography angiography, Atherosclerotic, Coronary artery plaque

## Abstract

**Background:**

The abnormal low-density protein cholesterol (LDL-C) level in the development of atherosclerosis is often comorbid in individuals with type 2 diabetes mellitus(T2DM). This study aimed to investigate the aggravating effect of abnormal LDL-C levels on coronary artery plaques assessed by coronary computed tomography angiography (CCTA) in T2DM.

**Materials and methods:**

This study collected 3439 T2DM patients from September 2011 to February 2022. Comparative analysis of differences in coronary plaque characteristics was performed for the patients between the normal LDL-C level group and the abnormal LDL-C level group. Factors with *P* < 0.1 in the univariable linear regression analyses were included in the multivariable linear stepwise regression.

**Results:**

A total of 2820 eligible T2DM patients were included and identified as the normal LDL-C level group (*n* = 973) and the abnormal LDL-C level group (*n* = 1847). Compared with the normal LDL-C level group, both on a per-patient basis and per-segment basis, patients with abnormal LDL-C level showed more calcified plaques, partially calcified plaques, low attenuation plaques, positive remodellings, and spotty calcifications. Multivessel obstructive disease (MVD), nonobstructive stenosis (NOS), obstructive stenosis (OS), plaque involvement degree (PID), segment stenosis score (SSS), and segment involvement scores (SIS) were likely higher in the abnormal LDL-C level group than that in the normal LDL-C level group (*P* < 0.001). In multivariable linear stepwise regression, the abnormal LDL-C level was validated as an independent positive correlation with high-risk coronary plaques and the degree and extent of stenosis caused by plaques (low attenuation plaque: β = 0.116; positive remodelling: β = 0.138; spotty calcification: β = 0.091; NOS: β = 0.427; OS: β = 0.659: SIS: β = 1.114; SSS: β = 2.987; PID: β = 2.716, all P value < 0.001).

**Conclusions:**

Abnormal LDL-C levels aggravate atherosclerotic cardiovascular disease (ASCVD) in patients with T2DM. Clinical attention deserves to be caught by the tailored identification of cardiovascular risk categories in T2DM individuals and the achievement of the corresponding LDL-C treatment goal.

**Supplementary Information:**

The online version contains supplementary material available at 10.1186/s12933-024-02304-0.

## Background

Cardiovascular disease (CVD) steadily represents the main cause of death in type 2 diabetes mellitus(T2DM) patients and is disproportionately affected by T2DM [1, 2]. T2DM has been identified as an established important risk factor in accelerating the development of atherosclerotic CVD (ASCVD) [3]. Dyslipidemia, classified as a condition characterized by an imbalance of atherogenic and protective lipids, especially an elevated plasma concentration of low-density lipoprotein cholesterol (LDL-C), is a major risk factor for ASCVD [4, 5]. It is worth drawing attention to the prevalence of elevated LDL-C among patients with T2DM has been as high as 50-68% [6–8].

Management strategies for diabetic dyslipidemia have significantly changed over recent years with the advent of newer lipid-lowering drugs [9]. Germane to diabetes, the 2019 European Society of Cardiology/European Atherosclerosis Society (ESC/EAS) Guideline for the Management of Dyslipidemias revised CV risk stratification and proposed new LDL-C goals. For diabetic patients at very high risk, an LDL-C goal of < 1.4 mmol/L is recommended; for diabetic patients at high risk, it is < 1.8 mmol/L; and for diabetic patients at moderate risk, it is < 2.6 mmol/L [10]. Despite the incremental interventions in recent years, diabetic patients have experienced a significant reduction in LDL-C, and many patients still do not achieve the ideal LDL-C goal [11].

Coronary computed tomography angiography (CCTA) allows for a noninvasive, detailed anatomic investigation of the coronary circulation and directly visualizes coronary atherosclerosis, and has become increasingly used for patients with ASCVD [12]. The population with T2DM demonstrated a high prevalence of high-risk coronary plaques and obstructive ASCVD as assessed by CCTA [13, 14]. Moreover, Cheng et al. [15] demonstrated that LDL-C was associated with an increased mixed plaque and non-calcified plaque burden in patients without previous ASCVD. Hirai et al.’s study [16] using CCTA analysis reported that even the achievement of the previous guideline-defined LDL-C target (< 1.8 mmol/L) would increase the calcified plaque volume and suppress the increase in low attenuation plaque volume in high-risk patients with acute coronary syndrome (ACS).

Abnormal LDL-C levels in the development of atherosclerosis are often comorbid in individuals with T2DM [17, 18]. However, little are known about the association of abnormal LDL-C levels after risk stratification with CCTA manifestations of coronary atherosclerotic plaque in various cardiovascular risk categories of T2DM patients. Therefore, this study aimed to stratify the cardiovascular risk of T2DM patients individually according to the new guidelines, determine whether their LDL-C levels were above or below the recommended thresholds for the corresponding risk level, assess the coronary plaque morphological characteristics in T2DM patients by CCTA, and explore the association between abnormal LDL-C levels and CCTA characteristics. Finally, determine whether abnormal LDL-C levels aggravate coronary atherosclerotic plaques in T2DM patients.

## Methods

### Study population

This study collected 3439 T2DM patients who underwent CCTA (from September 2011 to February 2022). Under the approval of our institution’s Biomedical Research Ethics Committee, informed consent was waived due to the retrospective nature of this investigation. T2DM conformed to the American Diabetes Association guidelines or was treated currently through oral glucose-lowering agents or insulin [19]. Exclusion criteria were: missing imaging files, serious artifacts, or poor image quality for evaluation; coronary artery bypass grafting, stent implantation or prosthetic valve replacement prior to CCTA examination; coronary artery fistula; severe renal failure [estimated glomerular filtration rate (eGFR) < 30 mL/min/1.73 m^2^]; and diagnosed neuroendocrine tumor. The clinical baseline data of all patients were recorded in detail (e.g., age, sex, past medical history, laboratory results). Regardless of whether they had quit smoking or not, they were all recorded as having a history of tobacco use, similar for the history of alcohol consumption. Patients who were treated for diabetes-related retina/ophthalmopathy, nephropathy, pedopathy, peripheral neuropathy, or significant plaque on carotid artery ultrasound or who otherwise had a prior diagnosis for these conditions were categorized as having that risk factor.

Patients were stratified into different risk categories (very-high risk, high risk, moderate risk, and low risk) based on various factors, including T2DM status and the presence of other cardiovascular risk factors in accordance with the 2019 ESC/EAS Guidelines for the Management of Dyslipidemia [10]. The recommended thresholds for normal LDL-C in T2DM patients: for very-high-risk patients, an LDL-C threshold of<1.4 mmol/L (<55 mg/dL); for high-risk patients, an LDL-C threshold of<1.8 mmol/L (<70 mg/dL); for moderate-risk patients, an LDL-C threshold of<2.6 mmol/L (<100 mg/dL); while low-risk patients aimed for<3.0 mmol/L (<116 mg/dL), according to the 2019 ESC/EAS Guidelines. Patients were further divided into a normal LDL-C level group and an abnormal LDL-C level group based on whether their LDL-C levels were below or above the recommended thresholds for their respective risk categories [10]. Dyslipidemia was diagnosed as consistent with at least one of the following conditions: hypercholesterolemia (TC ≥ 6.2 mmol/L), hyper-LDL-C (LDL-C ≥ 4.1 mmol/L), hypertriglyceridemia (TG ≥ 2.3 mmol/L), and hypo-HDL-C (HDL-C < 1.0 mmol/L in men and < 1.3 mmol/L in women) [4, 20].

### Coronary CTA obtain and analysis

#### Coronary CTA scanning protocols

All CCTA examinations for all enrolled T2DM patients were performed by scanning multidetector CT systems (SOMATOM Definition, Siemens Medical Solutions, Forchheim, Germany; and SOMATOM Definition FLASH, Siemens Medical Solutions, Forchheim, Germany, or a Revolution CT (GE Healthcare, Waukesha, WI, USA). During this process β receptor blockers were not used to lower heart rate. The range of coronary artery scans on patients whose hands were on either side of the head in a supine position from the upper tracheal bifurcation to the lower 20 mm below the inferior cardiac apex. A 70–90-mL {dependent on the body mass index (BMI)} bolus of iodinated contrast agent (iopamidol, 370 mg of iodine/mL) was injected into the antecubital vein at a flow rate of 5 mL/s and was followed by a bolus chaser of 30 mL of saline chaser at the same rate. For the SOMATOM Definition system, scanning was performed in conventional helical mode at a tube voltage of 100-120kVp, tube current of 220 mAs, collimation of 64/128 × 0.6 mm, and rotation time, of 0.33–0.4 s. For the Revolution CT system, the tube voltage and tube current were set automatically by kV Assist and Smart-mA based on the scout image of the patients, collimation was 256 × 0.625 mm, and the rotation time was 0.28 s. Retrospective electrocardiographic gating or a prospective electrocardiographic gating protocol was adopted to eliminate artifacts of cardiac motion. Multiple alternative reconstruction algorithms for revealing coronary artery plaques, including maximum intensity projections, multiplanar reconstructions, curvature plane reconstructions and volume reconstructions.

### CCTA characteristics analysis

The CCTA images of all involved T2DM patients were transferred to an image post- processing workstation (Syngo-Imaging, Siemens Medical Solution Systems, Forchheim, Germany) for visual evaluation. All visual evaluations of CCTA images were performed by three experienced cardiothoracic radiologists (J.Y.N., a cardiothoracic radiologist with four years of experience; G.Y., a cardiothoracic radiologist with eight years of experience; L.Y., a cardiothoracic radiologist with 18 years of experience) blinded to the clinical findings and group characteristics. The following coronary plaque characteristics assessed by CCTA were recorded, and the discrepancies in the observation between the three radiologists were resolved by consensus. The four branches of the coronary artery tree were composed of the left main (LM), left anterior descending (LAD), left circumflex (LCX), and right coronary artery (RCA), and the 16 segments were segmented based on modified American Heart Association report [21]. The severity of lumen stenosis caused by detected plaques was quantified and graded as a 5-point scale based on the Coronary Artery Disease(CAD)-Reporting and Data System [22]: Grade 0 (no visible plaque); Grade 1-minimal (1–24% luminal stenosis); Grade 2-mild (25–49% luminal stenosis); Grade 3-moderate (50–69% luminal stenosis); Grade 4-severe (70–99% luminal stenosis); Grade 5 (totally occluded). The plaque length score was defined as the percentage of plaque length covering the length of each segment, graded using a 4-point scale: Grade 0(no visible plaque), Grade 1-minimal (≤ 25% segment involvement); Grade 2-mild (26–50% segment involvement); Grade 3-moderate (51–75% segment involvement); Grade 4-severe (76–100% segment involvement.). The plaque involvement degree (PID) was the sum of the plaque length score for all segments (0–64). The segment involvement score (SIS) was defined as the number of coronary artery segments observed with plaques for each patient (0–16). The segment stenosis score (SSS) was defined as the sum of the stenosis scores of the relevant stenosis grades of all segments for each patient (0–80).

Coronary plaque refers to a lesion with an area of at least 1-mm^2^ on the surface or within the artery lumen, clearly distinctive from the vessel lumen. Plaques were visually classified as calcified plaque (CT attenuation of plaque higher than contrast-enhanced coronary lumen), non-calcified plaque (CT density of plaque lower than contrast-enhanced lumen without any calcification) and partially calcified plaque (both calcified and non-calcified components present in a single plaque) [23]. The high-risk coronary plaques included low attenuation plaque, positive remodelling, spotty calcification, napkin-ring sign. Low attenuation plaque was defined as CT values < 30 HU measured in an area > 1-mm^2^ within the plaque. The remodelling index was defined as the ratio between the maximum vessel diameter (including plaque and lumen) of the lesion segment and the normal proximal lumen diameter (arterial remodeling index = lesion plaque area/reference area), with a remodelling index ≥ 1.1 indicating positive remodelling. Spotty calcification was defined as any discrete calcification with a length diameter of < 3 mm in any plane within a non-calcified plaque, with a length diameter less than 1.5 times the vessel diameter and a short diameter of less than 2/3 the vessel diameter. The napkin-ring sign was described as a specific pattern of atherosclerotic plaques characterized by a plaque core with low CT attenuation surrounded by a rim-like area of higher CT attenuation [23–25]. Any stenosis greater than 50% was defined as obstructive stenosis (OS), and nonobstructive stenosis (NOS)was defined as without any OS. Multivessel obstructive disease (MVD) was defined as the presence of more than one vessel with stenosis ≥ 70% or LM stenosis ≥ 50%; this definition was proposed for myocardial revascularization as a marker of high-risk coronary artery disease by the American College of Cardiology/American Heart Association guidelines [26].

### Intra- and inter-observer reproducibility

The reproducibility of coronary plaque characteristics was calculated using intra- and inter-observer correlation coefficients (ICCs). A radiologist (J.Y.N) evaluates all coronary artery images. Then, after at least 30 days, the same radiologist, and another radiologist (G.Y.) independently reevaluated the randomly selected sequence of 150 CCTA images. Intra-observer reproducibility was assessed by comparing the radiologist one. The inter-observer reproducibility was evaluated by comparing the two radiologists.

### Statistical analysis

The Kolmogorov-Smirnov tests evaluated whether continuous variables were normally distributed. The Student’s *t*-tests were used for normally distributed continuous variables, and Mann–Whitney U tests were used for nonnormally distributed variables. Pearson *Χ*^2^ test for categorical variables and Fisher’s exact test. The clinical baseline and coronary plaque characteristics were summarized as the mean ± standard deviation for continuous variables and as counts (percentages, %) for categorical variables. Univariate linear regression analysis was performed to explore associations between abnormal LDL-C levels and CCTA characteristics on a per-segment basis. Factors with *P* < 0.1 in the univariable analyses were included in the multivariable linear stepwise regression model to identify the effect of abnormal LDL-C levels on coronary atherosclerotic plaque in T2DM patients. A two-tailed P value < 0.05 indicated statistical significance. All statistical analyses were performed using SPSS (version 26.0, IBM, Armonk, NY, USA). The production of all bar charts and forest plots was completed using GraphPad Prism (version 9.5.0).

## Results

### Demography and clinical baseline characteristics of patients

A total of 2820 T2DM patients who matched the conditions were included in the final analysis, and were predominantly males (62.70%), with a mean age of 68.35 ± 10.52 years and a mean BMI of 24.63 ± 3.18 kg/m^2^. Nine hundred seventy-three patients were identified as normal LDL-C level group, and 1847 patients were identified as the abnormal LDL-C level group. The clinical baseline characteristics of the study cohorts are listed in Table 1.

**Table 1:** Clinical baseline characteristics of T2DM patients

Characteristic	Total (*n* = 2820)	T2DM		*P* value
		Normal LDL-C level (*n* = 973)	Abnormal LDL-C level (*n* = 1847)	
Age, years	68.35 ± 10.52	67.83 ± 10.81	68.62 ± 10.36	0.044
BMI (kg/m^2^)	24.63 ± 3.18	24.54 ± 3.08	24.68 ± 3.22	0.430
Sex/Male, n (%)	1768 (62.70%)	625 (64.23%)	1143 (61.88%)	0.220
Blood Pressure (mmHg)				
SBP	137.22 ± 19.49	134.29 ± 18.82	138.77 ± 19.67	< 0.001
DBP	79.52 ± 11.97	78.26 ± 11.68	80.19 ± 12.07	< 0.001
Medical History, n (%)				
Hypertension	1934 (68.58%)	649 (66.70%)	1285 (69.57%)	0.118
Dyslipemia	1295 (45.92%)	467 (48.00%)	828 (44.83%)	0.109
Alcohol consumption	868 (30.78%)	285 (29.29%)	583 (31.56%)	0.214
Tobacco use	1107 (39.26%)	378 (38.85%)	729 (39.47%)	0.748
Myocardial infarction	36 (1.28%)	4 (0.41%)	32 (1.73%)	0.003
Lab Values				
HbA_1_c (%)	7.62 ± 1.37	7.50 ± 1.35	7.68 ± 1.38	< 0.001
TG (mmol/L)	1.67 ± 1.34	1.62 ± 1.71	1.70 ± 1.10	< 0.001
TC (mmol/L)	4.07 ± 1.13	3.32 ± 0.90	4.47 ± 1.04	< 0.001
HDL‑ C(mmol/L)	1.14 ± 0.34	1.08 ± 0.35	1.16 ± 0.34	0.030
LDL‑ C (mmol/L)	2.32 ± 0.92	1.63 ± 0.61	2.68 ± 0.84	< 0.001
eGFR (ml/min/1.73m^2^)	81.74 ± 18.06	83.90 ± 17.72	80.60 ± 18.13	< 0.001
Symptoms, n (%)				
Unstable angina	43 (1.52%)	7 (0.72%)	36 (1.95%)	0.011
Stable angina	9 (0.32%)	1 (0.10%)	8 (0.43%)	0.139
Stroke/TIA	26 (0.92%)	6 (0.62%)	20 (1.08%)	0.218
Target organ damage, n (%)				
Eye/retina	104 (3.69%)	31 (3.19%)	73 (3.95%)	0.305
Kidney	97 (3.44%)	39 (4.01%)	58 (3.14%)	0.229
Foot	78 (2.77%)	18 (1.85%)	60 (3.25%)	0.031
Peripheral nerves	344 (12.20%)	118 (12.13%)	226 (12.24%)	0.933
Carotid artery	419 (14.86%)	76 (7.81%)	343 (18.57%)	< 0.001
Antidiabetes medications, n (%)				
Biguanides	1121 (39.75%)	409 (42.03%)	712 (38.55%)	0.072
Sulfonylurea	548 (19.43%)	168 (17.27%)	380 (20.57%)	0.035
α-Glucosidase inhibitor	682 (24.18%)	239 (24.56%)	383 (20.74%)	0.020
GLP‑1/DPP‑4 inhibitor	160 (5.67%)	46 (4.73%)	104 (5.63%)	0.310
Insulin	807 (28.62%)	243 (24.97%)	564 (30.54%)	0.002
Diet control	74 (2.62%)	35 (3.60%)	39 (2.11%)	0.019
Other oral	121 (4.29%)	39 (4.01%)	82 (4.44%)	0.591
Antihyperlipidemic medications, n (%)				
Statins	512 (18.16%)	211 (21.69%)	301 (16.30%)	< 0.001
Fibrates	25 (0.89%)	5 (0.51%)	20 (1.08%)	0.143
Other oral	92 (3.26%)	29 (2.98%)	63 (3.41%)	0.579
Antihypertensive medications, n (%)				
AECI/ ARB	832 (29.50%)	288 (29.60%)	544 (29.45%)	0.936
β-blocker	375 (13.30%)	132 (13.57%)	243 (13.16%)	0.761
Calcium antagonist	1082 (38.37%)	363 (37.31%)	719 (38.93%)	0.400
Diuretic	233 (8.26%)	83 (8.53%)	150 (8.12%)	0.708
Other oral	22 (0.78%)	6 (0.62%)	16 (0.87%)	0.474
Other treatment, n (%)				
Aspirin	386 (13.69%)	137 (14.08%)	249 (13.48%)	0.660
Clopidogrel	180 (6.38%)	65 (6.68%)	115 (6.23%)	0.639
Nitrate	123 (4.36%)	43 (4.42%)	80 (4.33%)	0.913

Data are presented as mean ± SD and numbers (percentages)

BMI – body mass index, SBP, systolic blood pressure; DBP, diastolic blood pressure; HbA_1_c, glycated haemoglobin; TG, triglyceride, TC, total cholesterol, LDL-C Low density lipoprotein cholesterol, HDL-C High density lipoprotein cholesterol; eGFR, estimated glomerular filtration rate; TIA, transient ischemic attack, GLP-1/DPP-4 inhibitor, glucagon-like peptide-1/dipeptidyl peptidase 4 inhibitors; ACEI, angiotensin converting enzyme inhibitor; ARB, angiotensin receptor blocker

P value represents the result of comparison between subgroups

Compared with the normal LDL-C level group, patients with abnormal LDL-C levels were more likely to be older (*P* = 0.044), and have higher blood pressure (*P* < 0.001). The HbA_1_c, TG, TC, HDL‑C, LDL‑C and eGFR were lower in the normal LDL-C level group than in the abnormal LDL-C level group (*P* < 0.05). The abnormal LDL-C level group experienced more myocardial infarction and unstable angina (*P* < 0.05). Although there was no significant difference between the two groups, stable angina and stroke/TIA showed an increasing trend in the abnormal LDL-C level group. The abnormal LDL-C level group experienced more foot damage and carotid artery damage in the target organ (*P* < 0.05). In addition, most T2DM in the two groups received antidiabetes medications, of which the abnormal LDL-C level group used more sulfonylurea, insulin injection, and less α-glucosidase inhibitor and diet control (*P* < 0.05). More patients in the normal LDL-C level group received statins for antihyperlipidemic medications than those in the abnormal LDL-C level group (*P* < 0.001).

## Comparative analysis of coronary plaque characteristics assessed by CCTA between the normal LDL-C level group and the abnormal LDL-C level group on a per-patient basis

**Fig. 1:**
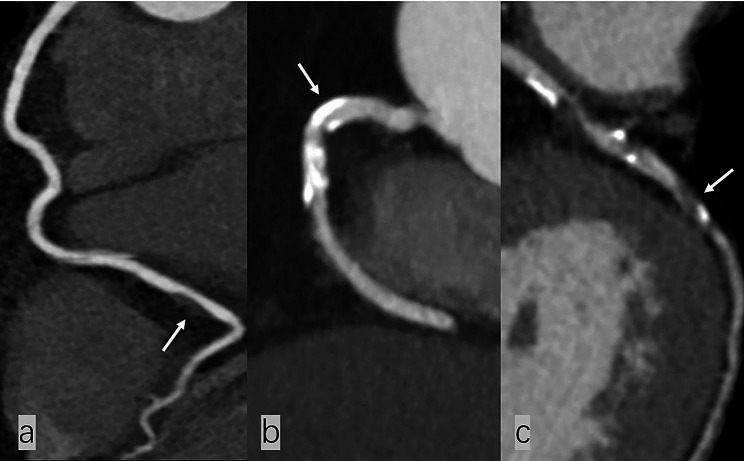
Image of coronary atherosclerotic plaque types. Non-calcified plaque (**a**); calcified plaque (**b**); partially calcified plaque (**c**)

**Fig. 2:**
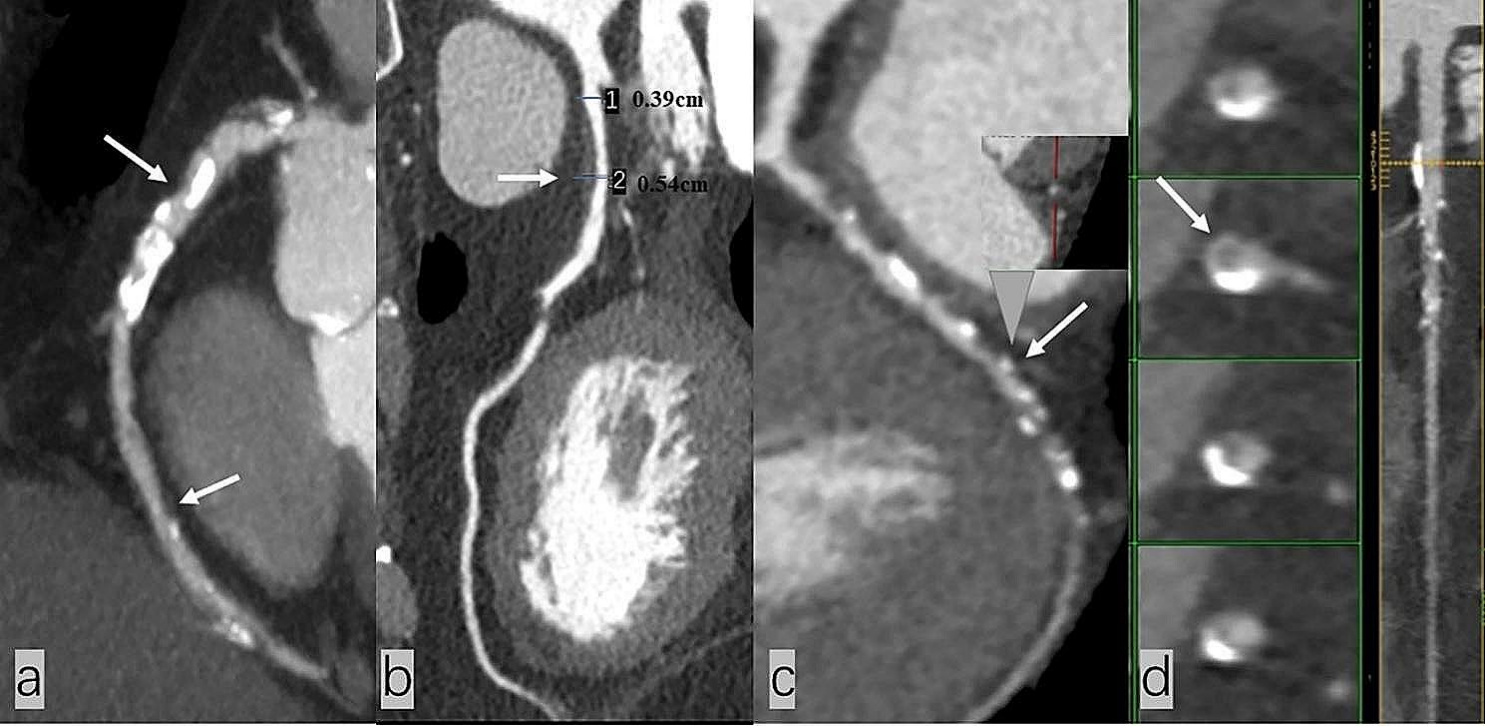
Image of high-risk coronary plaques. Low attenuation plaque in a 79-year-old male T2DM patients with abnormal LDL-C level (**a**); positive remodelling with a remodelling index = 1.38 {lesion plaque area (0.54 cm)/ reference area (0.39 cm)} in an 81-year-old male T2DM patients with abnormal LDL-C level (**b**); spotty calcification in a 54-year-old male T2DM patients with normal LDL-C level (**c**); napkin-ring sign in a 79-year-old male T2DM patients with abnormal LDL-C level (**d**)

**Fig. 3:**
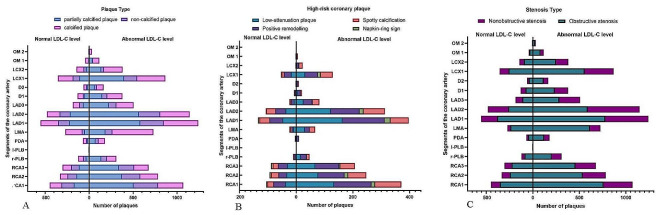
Visualization of the distribution of three types of plaques (**a**), high-risk plaques (**b**), and luminal stenosis caused by plaques (**c**) in coronary artery tree segments

On a per-patient basis, patients with abnormal LDL-C levels showed more calcified plaques (1153, 62.43% vs. 515, 52.93%, *P* < 0.001) and partially calcified plaques (1121, 60.69% vs. 453, 46.56%, *P* < 0.001). Coronary atherosclerotic plaque types are shown in Fig. 1. Compared with the normal LDL-C level group, low attenuation plaques, positive remodellings, and spotty calcifications in high-risk coronary plaques were higher in the abnormal LDL-C level group (379, 20.52% vs. 148, 15.21%, *P* = 0.001; 442, 23.93% vs. 155, 15.93%, *P* < 0.001; 282, 15.27% vs. 112, 11.51%, *P* = 0.006, respectively). Napkin-ring signs were not different between the two groups (*P* = 0.176). Representative high-risk coronary plaques are shown in Fig. 2. The MVD in the abnormal LDL-C level group was significantly higher than in the normal LDL-C level group. The detailed analysis results are listed in Table 2. Visualization of the three types of plaques and high-risk plaques in the coronary tree segments is shown in Fig. 3.

**Table 2:** Coronary plaque characteristics assessed by CCTA in T2DM patients with normal LDL-C level or abnormal LDL-C level

CCTA Characteristic	Total (*n* = 2820)	T2DM		*P* value
		Normal LDL-C level (*n* = 973)	Abnormal LDL-C level (*n* = 1847)	
**Per-patient**				
Plaque type, n (%)				
Any calcified plaque	1668 (59.15%)	515 (52.93%)	1153 (62.43%)	< 0.001
Any non-calcified plaque	1356 (48.09%)	448 (46.04%)	908 (49.16%)	0.115
Any partially calcified plaque	1574 (55.82%)	453 (46.56%)	1121 (60.69%)	< 0.001
High-risk Coronary Plaque Type, n (%)				
Low attenuation plaque	527 (18.69%)	148 (15.21%)	379 (20.52%)	0.001
Positive remodelling	597 (21.17%)	155 (15.93%)	442 (23.93%)	< 0.001
Spotty calcification	394 (13.97%)	112 (11.51%)	282 (15.27%)	0.006
Napkin-ring sign	74 (2.62%)	20 (2.06%)	54 (2.92%)	0.176
No. of plaque involved segments, n (%)				< 0.001
0	400 (14.18%)	195 (20.04%)	205 (11.10%)	
1	328 (11.63%)	129 (13.26%)	199 (10.77%)	
2	329 (11.67%)	135 (13.87%)	194 (10.50%)	
3	293 (10.39%)	86 (8.84%)	207 (11.21%)	
≥ 4	1470 (52.13%)	428 (43.99%)	1042 (56.42%)	
MVD	245(8.69%)	53 (5.45%)	192 (10.40%)	< 0.001
**Per-segment**				
Plaque type				
Calcified plaques	1.59 ± 2.01	1.42 ± 1.99	1.67 ± 2.02	< 0.001
Non-calcified plaques	0.89 ± 1.23	0.83 ± 1.20	0.92 ± 1.24	0.056
Partially calcified plaques	1.82 ± 2.46	1.37 ± 2.14	2.05 ± 2.58	< 0.001
High-risk coronary plaque				
Low attenuation plaque	0.33 ± 0.84	0.25 ± 0.71	0.37 ± 0.90	< 0.001
Positive remodelling	0.33 ± 0.78	0.24 ± 0.67	0.38 ± 0.82	< 0.001
Spotty calcification	0.22 ± 0.64	0.16 ± 0.51	0.25 ± 0.70	0.004
Napkin-ring sign	0.04 ± 0.27	0.03 ± 0.22	0.04 ± 0.29	0.166
Segments				
NOS	2.87 ± 2.25	2.56 ± 2.27	3.03 ± 2.23	< 0.001
OS	1.42 ± 2.28	1.06 ± 1.98	1.61 ± 2.39	< 0.001
PID	8.71 ± 8.71	7.08 ± 8.01	9.57 ± 8.94	< 0.001
SSS	9.18 ± 9.05	7.43 ± 8.27	10.10 ± 9.31	< 0.001
SIS	4.29 ± 3.36	3.62 ± 3.24	4.65 ± 3.38	< 0.001

Data are presented as mean ± SD and numbers (percentages)

MVD, multivessel obstructive disease; NOS, nonobstructive stenosis; OS, obstructive stenosis; PID: plaque involvement degree; SSS, segment stenosis score; SIS: segment involvement score

P value represents the result of comparison between subgroups

## Comparative analysis of coronary plaque characteristics assessed by CCTA between the normal LDL-C group and the abnormal LDL group on a per-segment basis

On per-segment basis, patients with abnormal LDL-C level showed more calcified plaques (1.67 ± 2.02 vs. 1.42 ± 1.99, *P* < 0.001) and partially calcified plaques (2.05 ± 2.58 vs. 1.37 ± 2.14, *P* < 0.001). Compared with the normal LDL-C level group, low attenuation plaques, positive remodellings, and spotty calcifications in high-risk coronary plaque were higher in the abnormal LDL-C level group (0.37 ± 0.90 vs. 0.25 ± 0.71, *P* < 0.001; 0.38 ± 0.82 vs. 0.24 ± 0.67, *P* < 0.001; 0.25 ± 0.70 vs. 0.16 ± 0.51, *P* = 0.004, respectively). The napkin-ring signs were not different between the groups (*P* = 0.166). NOS, OS, PID, SSS, and SIS were likely higher in the abnormal LDL-C level group than in the normal LDL-C level group (*P* < 0.001). The detailed analysis results are listed in Table 2. Visualization of the luminal stenosis caused by plaques in the coronary tree segments is shown in Fig. 3.

## Aggravating the effect of abnormal LDL-C on the degree of stenosis and involvement of coronary artery plaques in T2DM patients

**Fig. 4:**
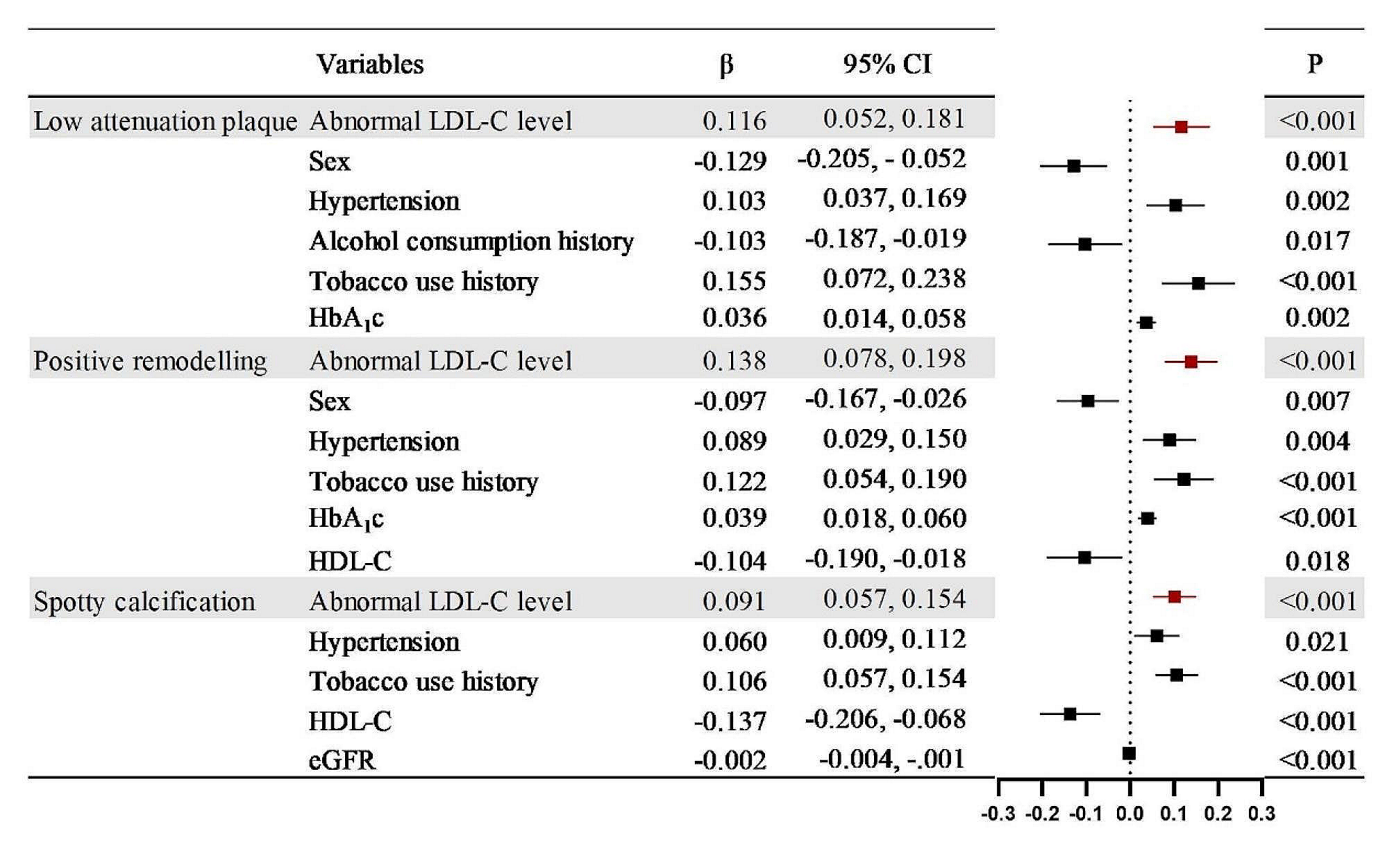
Multivariate linear stepwise regression analysis of the aggravating effect of abnormal LDL-C levels and confounding factors on high-risk plaques in T2DM patients. β is the multivariable linear stepwise regression coefficient. CI, Confidence interval. Abbreviations are consistent with Table 2

**Fig. 5:**
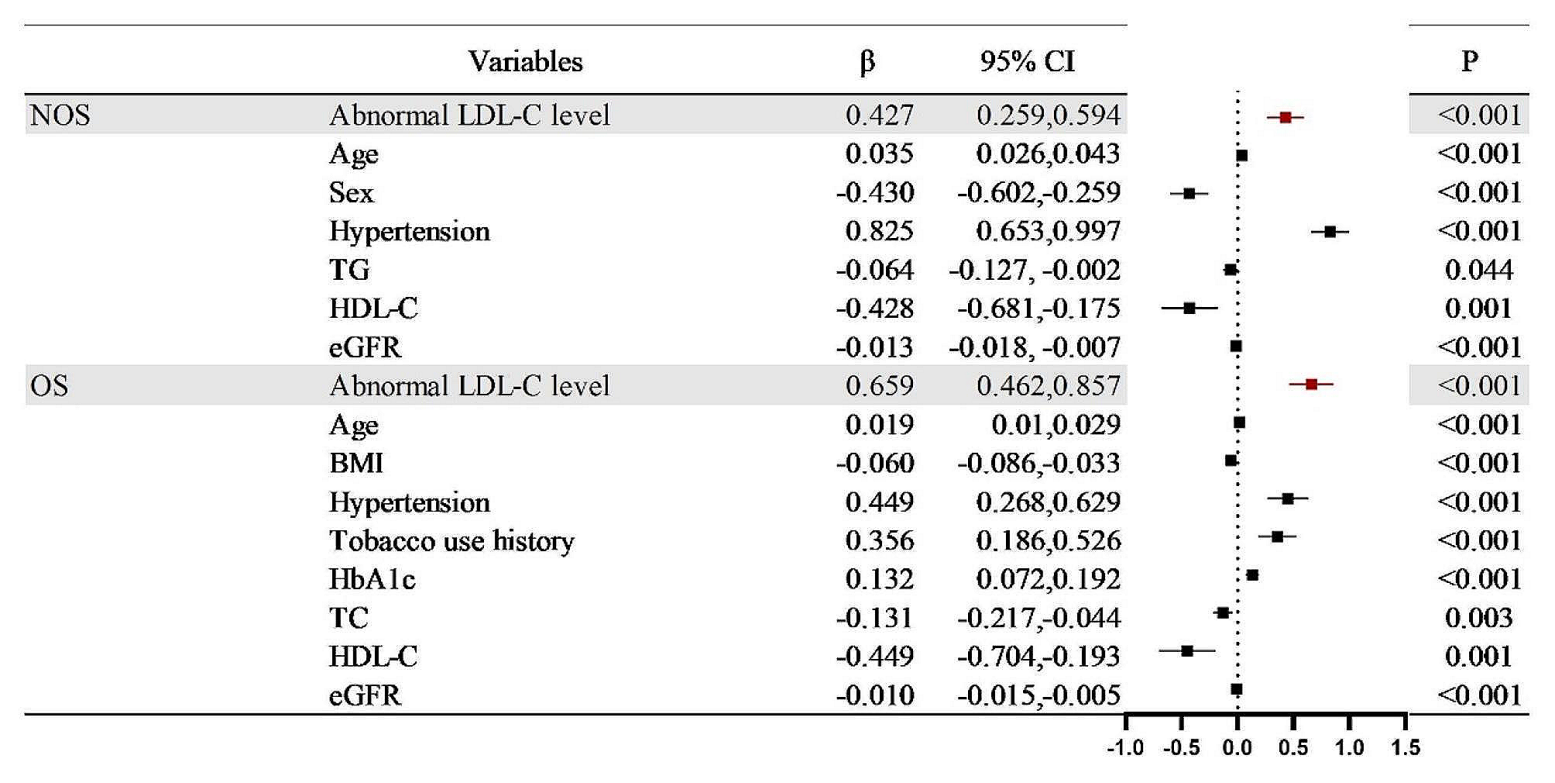
Multivariate linear stepwise regression analysis of the aggravation effect of abnormal LDL-C levels and confounding factors on NOS and OS in T2DM patients. Abbreviations are consistent with Table 2; Fig. 4

**Fig. 6:**
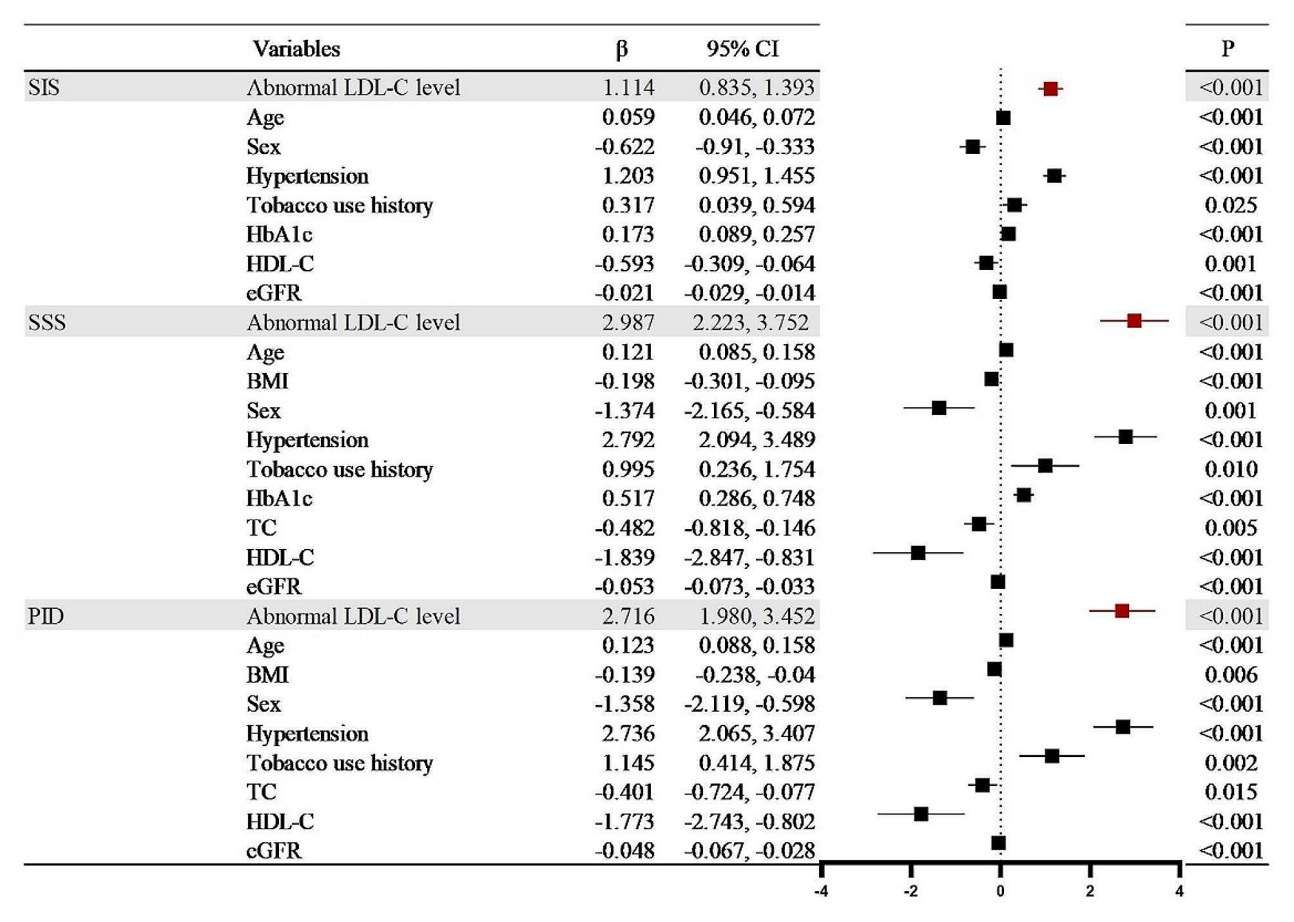
Multivariate linear stepwise regression analysis of the aggravating effect of abnormal LDL-C levels and confounding factors on the SIS, SSS and PID in T2DM patients. Abbreviations are consistent with Table 2; Fig. 4

Univariate linear regression analysis of abnormal LDL-C levels and high-risk coronary plaques in T2DM patients revealed positive correlations between abnormal LDL-C levels with low attenuation plaques (*r* = 0.121), positive remodellings (*r* = 0.138), and spotty calcifications (*r* = 0.090). All *P* < 0.1. Positive correlations were also presented between abnormal LDL-C levels and NOS (*r* = 0.470), OS (*r* = 0.551), SIS (*r* = 1.021), SSS (*r* = 2.672), and PID (*r* = 2.490). All of them had a P value < 0.1(Table 3). In multivariable linear stepwise regression, the abnormal LDL-C level was validated as an independent positive correlation with high-risk coronary plaques and the degree and extent of stenosis caused by plaques (low attenuation plaque: β = 0.116; positive remodelling: β = 0.138; spotty calcification: β = 0.091; NOS: β = 0.427; OS: β = 0.659: SIS: β = 1.114; SSS: β = 2.987; PID: β = 2.716, all P value < 0.001) (Figs. 4, 5 and 6).

**Table 3:** Univariate linear regression analysis of aggravating effect of abnormal LDL– C level on coronary plaques in T2DM patients

Clinical baseline characteristics	Low attenuation plaques	Positive remodelling	Spotty calcification	NOS	OS	SIS	SSS	PID
	r	r	r	r	r	r	r	r
Abnormal LDL– C level	0.121^*§^	0.138^*§^	0.090^*§^	0.470^*§^	0.551^*§^	1.021^*§^	2.672^*§^	2.490^*§^
Age, years	– 0.001	– 0.003^§^	0.002^§^	0.047^*§^	0.030^*§^	0.077^*§^	0.172^*§^	0.165^*§^
BMI	– 0.004	– 0.006	– 0.003	0.024^§^	– 0.048^*§^	– 0.023	– 0.144^*§^	– 0.090^§^
Sex (female)	– 0.164^*§^	– 0.180^*§^	– 0.101^*§^	– 0.320^*§^	– 0.331^*§^	– 0.656^*§^	– 1.713^*§^	– 1.760^*§^
Hypertension	0.095^*§^	0.082^*§^	0.073^*§^	1.021^*§^	0.545^*§^	1.562^*§^	3.420^*§^	3.364^*§^
Dyslipemia	0.044	0.053^§^	0.074^*§^	0.028	0.102	0.127	0.438	0.452
Alcohol consumption	0.059^§^	0.124^*§^	0.032	– 0.003	0.046	0.047	0.168	0.310
Tobacco use	0.166^*§^	0.185^*§^	0.112^*§^	0.123	0.320^*§^	0.448^*§^	1.351^*§^	1.487^*§^
HbA1c	0.042^*§^	0.047^*§^	0.011	0.028	0.133^*§^	0.162^*§^	0.511^*§^	0.500^*§^
TG	0.027^*§^	0.020^§^	– 0.003	– 0.079^*§^	– 0.046	– 0.125^*§^	– 0.266^*§^	– 0.215^§^
TC	0.009	0.015	– 0.003	– 0.075^*§^	– 0.068^§^	– 0.145^*§^	– 0.320^*§^	– 0.289^*§^
HDL– C	– 0.130^*§^	– 0.157^*§^	– 0.142^*§^	– 0.275^*§^	– 0.361^*§^	– 0.634^*§^	– 1.693^*§^	– 1.720^§^
LDL‑C	0.008	0.018	0.013	– 0.021	– 0.045	– 0.069	– 0.154	– 0.132
eGFR	– 5.922E-5	7.129E-5	– 0.003^*§^	– 0.026^*§^	– 0.018^*§^	– 0.044^*§^	– 0.100^*§^	– 0.095^*§^

The r is the univariate linear regression coefficient. Abbreviations are consistent with Tables 1 and 2. Factors with *P* < 0.1 in the univariable analyses were included in the stepwise multiple linear model

^*^P < 0.05

^§^*P* < 0.1

### Intra- and inter-observer reproducibility

There is excellent intra- and inter-observer reproducibility for the severity of lumen stenosis and plaque length score both intra- and inter-observer correlation coefficients, ranging from 0.957 to 0.978; for low attenuation plaque, positive remodelling, spotty calcification, and napkin-ring sign, ranging from 0.799 to 0.958. The detailed results of ICCs are listed in Supplementary material s-Table 1.

## Discussion

This study stratified the cardiovascular risk categories of T2DM patients individually and then investigated the aggravating effect of abnormal LDL-C levels on coronary artery plaques assessed by CCTA in T2DM. The main findings are as follows: (i) compared with normal LDL-C level patients, more calcified plaques and partially calcified plaques, and more high-risk plaques were detected in comorbid abnormal LDL-C level and T2DM patients; (ii) when abnormal LDL-C level was combined, the degree of stenosis caused by coronary atherosclerotic plaque in T2DM patients was more serious and the involvement was more extensive; (iii) our analysis also suggested that abnormal LDL-C level was an independent positive correlation factor after adjustment for confounding factors that aggravated high-risk plaques, degree of stenosis, and involvement in patients with T2DM. Moreover, abnormal LDL-C levels presented a stronger correlation than other independently correlated factors in multivariate regression equations.

### The aggravating effect of abnormal LDL-C levels on plaque type and high-risk plaques

T2DM patients present greater atherosclerotic plaque burden and unique plaque composition characterized by complexity [27, 28]. Gao et al. [29] found that a larger plaque burden and a higher percentage of calcified plaque and partially calcified plaque were observed in diabetic patients than in nondiabetic patients. They thought T2DM patients with CVD have a predominant partially calcified plaque on CCTA, possibly indicating a high risk for plaque rupture and hospital admission for ACS. Similar results were obtained in our study; we also found that the prevalence of some symptoms and some target organs increased to varying degrees in the abnormal LDL-C level group, and more calcified plaques and partially calcified plaques were observed in T2DM patients with abnormal LDL levels than in T2DM patients with normal LDL-C levels. Another noteworthy point. In a long-term observational study, all asymptomatic T2DM patients with baseline LDL-C levels ≥ 1.8mmol developed ACS during a later follow-up, and there was a clear relationship between a low attenuation plaque volume > 10% of total plaque and LDL-C level (*P* = 0.012) [30]. Tanaka et al. [31] conducted a prospective study on 70 patients with T2DM. The results showed that an increase in LDL-C was significantly correlated with an increase in the per cent change in necrotic core volume (*P* = 0.03). Unfortunately, a larger necrotic core characterizes vulnerable plaques and may cause plaque rupture. In a previous study, Burke et al. [32] had already proposed that the highest remodelling index was calculated at the point of plaque rupture. Finn et al. [33] concluded that all lesions derived from or related to plaque rupture show positive remodelling, which may represent one important surrogate for detecting lesion vulnerability. Our study showed a significant independent positive association between abnormal LDL-C levels and low attenuation plaque, positive remodelling, and spotty calcification. More positive remodellings were observed in the abnormal LDL-C level group compared to the normal LDL-C level group.

A previous study investigating acute chest pain demonstrated the prevalence of napkin-ring signs in ACS patients was 32.4%, indicating that the napkin-ring sign indicates rupture-prone plaques [34]. In the stable patients with CAD, the prevalence of napkin-ring signs in our study was similar to that of no-ACS patients in previous CCTA studies [35, 36]. No statistical difference in napkin-ring signs between the two groups might indicate that our study population did include basically stable CAD with T2DM.

Considering the above, these CCTA observations could suggest that ASCVD develops more rapidly in the presence of abnormal LDL-C levels and that abnormal LDL-C levels may further exacerbate the risk of coronary artery plaque rupture and ACS in T2DM patients requiring additional clinical attention.

## The aggravating effect of abnormal LDL-C levels on the degree of stenosis and range of involvement

In a previous study by Deseive et al. [37] investigated the effect of diabetes on total coronary plaque volume, and they found that diabetic patients with more than 110.5 mm³ of coronary artery total plaque volume had higher LDL by trend compared to diabetic patients with less than 110.5 mm³. Our study reported similar results: In T2DM patients, abnormal LDL-C levels were positively correlated with indicators such as SIS, SSS, and PID, which represent the degree of stenosis and involvement of coronary artery plaques. These data suggest that with the presence of abnormal LDL-C levels, the degree of coronary artery stenosis is more severe and the range of involvement is wider. The first observable change for plaque formation is the aggregation of LDL-C in the intima, and monocytes transmigrate across into the intima, where they proliferate, differentiate into macrophages, and take up the highly oxidized LDL-C, forming foam cells. Which later result in atherosclerotic plaque formation and enlargement. The lesions grow towards the adventitia until a critical point is reached, after which they expand outwards and invade the lumen [38]. Importantly, elevated small and dense LDL particles are one of the characteristics of dyslipidemia in T2DM. In the process of T2DM, insulin resistance can cause an increase in free fatty acids in the serum, thereby driving an increase in these smaller LDL-C particles [39]. Small and dense LDL-C particles, probably contribute to accelerated atherosclerosis even before diabetes is formally diagnosed [40].

## The independent positive association of abnormal LDL-C with the exacerbation of ASCVD

Previous studies typically used a specific cut-off value to determine abnormal LDL-C levels for generalized patients. However, this may weaken our consideration of the effect of other potential factors on CVD in T2DM patients. These factors may require reducing the LDL-C level of patients during ASCVD treatment, which may have greater potential benefits for patients [41]. Prevention of ASCVD in each person should relate to his or her total CV risk; the higher the risk is, the more intensive the reduction in LDL-C should be [10]. Fujihara et al. [42] reported that oxidized LDL-C could play key roles in the progression of atherosclerosis and was an independent predictor of coronary artery stenosis by CCTA in asymptomatic patients with T2DM. Our research results show that the abnormal LDL-C level after risk stratification not only exhibited an independent positive correlation with high-risk coronary plaques and the degree and extent of stenosis caused by plaques in multivariable linear stepwise regression but also had a higher coefficient of influence compared to other confounding factors, ranked in the first two positions. These unfavorable CCTA findings, such as severe stenosis or high-risk plaques, were established risk factors exactly for mortality and adverse cardiovascular events in T2DM patients [43]. Therefore, tailored identification of cardiovascular risk based on the specific condition of this patient and corresponding LDL-C target in diabetic patients is very important and deserves clinical attention.

## Limitation

Our study has some limitations. First, this is a single-center retrospective cross-sectional study that may lead to patient selection bias. We visually assessed coronary plaque in patients with diabetes undergoing CCTA without using any AI-assisted automatic or semiautomatic quantitative assessment. Finally, some potential confounding factors, such as exercise and dietary intake habits of high cholesterol and saturated fatty acids that may affect LDL-C levels, were not obtained and analysed in the study. Therefore, our results still need to be validated by more centers and more comprehensive prospective studies.

## Conclusions

In the presence of abnormal LDL-C levels, T2DM patients exhibit more high-risk plaques in the coronary arteries, the degree of coronary artery stenosis is more severe, and the range of involvement is wider. Abnormal LDL-C levels were strongly associated with ASCVD in patients with T2DM and were independently associated with some CCTA manifestations. Clinical attention deserves to be caught by the tailored identification of cardiovascular risk categories in specific T2DM individuals and the achievement of the corresponding LDL-C treatment goal.

### Electronic supplementary material

Below is the link to the electronic supplementary material.

Supplementary Material 1

## Data Availability

The datasets used and analyzed during the current study are available from the corresponding author on reasonable request.

## Abbreviations

T2DM: Type 2 diabetes mellitus
CCTA: Coronary computed tomography angiography
LDL-C: Low-density protein cholesterol
ASCVD: Atherosclerotic cardiovascular disease
LDL-C: Low density lipoprotein cholesterol
ESC/EAS: European Society of Cardiology/European Atherosclerosis Society
ACS: Acute coronary syndrome
TC: Total cholesterol
TG: Triglyceride
HDL-C: High density lipoprotein cholesterol
LM: Left main
LAD: Left anterior descending
LCX: Left circumflex
RCA: Right coronary artery
CAD: Coronary artery disease
PID: Plaque involvement degree
SIS: Segment involvement scores
OS: Obstructive stenosis
NOS: Nonobstructive stenosis
SSS: Segment stenosis score
MVD: Multivessel obstructive disease
