# Supplementary material for: Aggravating effect of abnormal low-density protein cholesterol level on coronary atherosclerotic plaque in type 2 diabetes mellitus patients assessed by coronary computed tomography angiography
Source: Cardiovasc Diabetol. 2024 Jul 4;23:234. doi: 10.1186/s12933-024-02304-0 (PMC11225366; doi:10.1186/s12933-024-02304-0)
Supplement: Supplementary file 1 — Supplementary Material 1 [file 12933_2024_2304_MOESM1_ESM.docx]

s-Table 1 Inter- and intra- observer reproducibility of coronary plaque characteristics

| Coronary plaque characteristics | Intra-observer correlation coefficients (95% CI) | Inter-observer correlation coefficients (95% CI) |
| --- | --- | --- |
| Severity of lumen stenosis | 0.961(0.953,0.967) | 0.957(0.949,0.965) |
| Plaque length score | 0.978(0.974,0.982) | 0.969(0.963,0.974) |
| Low attenuation plaque | 0.958(0.950,0.965) | 0.935(0.922,0.946) |
| Positive remodelling | 0.952(0.943,0.960) | 0.939(0.926,0.949) |
| Spotty calcification | 0.929(0.914,0.941) | 0.898(0.877,0.915) |
| Napkin-ring sign | 0.923(0.907,0.936) | 0.799(0.758,0.833) |

CI, Confidence interval.
